# A Land-Use Perspective for Birdstrike Risk Assessment: The Attraction Risk Index

**DOI:** 10.1371/journal.pone.0128363

**Published:** 2015-06-26

**Authors:** Francesca Coccon, Matteo Zucchetta, Giulia Bossi, Matteo Borrotti, Patrizia Torricelli, Piero Franzoi

**Affiliations:** 1 Department of Environmental Sciences, Informatics and Statistics, University Ca’ Foscari of Venice, Venice, Italy; 2 CNR-IRPI–National Research Council of Italy, Research Institute for Geo-Hydrological Protection, Padua, Italy; 3 European Centre for Living Technology, University Ca’ Foscari of Venice, Venice, Italy; 4 CNR-IMATI—National Research Council of Italy, Institute of Applied Mathematics and Information Technology, Milan, Italy; US Army Engineer Research and Development Center, UNITED STATES

## Abstract

Collisions between aircraft and birds, birdstrikes, pose a serious threat to aviation safety. The occurrence of these events is influenced by land-uses in the surroundings of airports. Airports located in the same region might have different trends for birdstrike risk, due to differences in the surrounding habitats. Here we developed a quantitative tool that assesses the risk of birdstrike based on the habitats within a 13-km buffer from the airport. For this purpose, we developed Generalized Linear Models (GLMs) with binomial distribution to estimate the contribution of habitats to wildlife use of the study area, depending on season. These GLMs predictions were combined to the flight altitude of birds within the 13-km buffer, the airport traffic pattern and the severity indices associated with impacts. Our approach was developed at Venice Marco Polo International airport (VCE), located in northeast Italy and then tested at Treviso Antonio Canova International airport (TSF), which is 20 km inland. Results from the two airports revealed that both the surrounding habitats and the season had a significant influence to the pattern of risk. With regard to VCE, agricultural fields, wetlands and urban areas contributed most to the presence of birds in the study area. Furthermore, the key role of distance of land-uses from the airport on the probability of presence of birds was highlighted. The reliability of developed risk index was demonstrated since at VCE it was significantly correlated with bird strike rate. This study emphasizes the importance of the territory near airports and the wildlife use of its habitats, as factors in need of consideration for birdstrike risk assessment procedures. Information on the contribution of habitats in attracting birds, depending on season, can be used by airport managers and local authorities to plan specific interventions in the study area in order to lower the risk.

## Introduction

Aircraft collisions with wildlife, wildlife strikes, pose a serious and growing threat to civil aviation safety [1, 2]. Most of these events are bird-aircraft collisions, known as birdstrikes, which are of major concern because of the impact frequency and associated damage [2, 3]. From 1988 to 2009, over 229 lives and 221 aircrafts (military and civil) were lost worldwide as a result of birdstrikes [4–7] and the estimated cost of the global civil aviation, considering direct and indirect expenses, is over U.S. $1.2 billion annually [8, 9]. Importantly, these birdstrike statistics reflect an increasing trend [10]. This is primarily due to the considerable increase of air traffic [11] and the demographic explosion of synanthropic species (e.g. gulls, rock doves, crows and starlings) [12], which have started to exploit human-modified habitats and anthropogenic food sources [13, 14]. As a consequence, land-uses in and around airports may affect wildlife abundance and distribution, influencing their daily and seasonal movements [15–19]. These factors result in an increased risk of birdstrike. In this paper, we have adopted the definition of risk proposed by Blackwell et al [20]. Risk is defined as the probability of damage to an aircraft posed by a species, if struck, and the probability of strike occurring. Notably, wildlife exploitation of land-uses is particularly hazardous for aviation in the vicinity of approach and departure zones (i.e. the Air Operations Area, AOA). By definition, a hazard represents a particular state or condition that can affect the probability of birdstrikes [20]. In fact, aircraft operating at low altitudes within the approach/departure zones may interfere with wildlife present in these areas leading to a possible harm [21]. However, 99% of birdstrikes occurs below 2000ft (= 609 m). An aircraft on a normal approach reaches this altitude approximately when at 13-km from the runway [22]. It follows that land-use practices within this radius from the airport may affect the risk of interference between aircraft and birds. For this reason, in recent years, the importance of considering land-use and land-cover management into risk assessment procedures, as well as the need to perceive airfields as an integral part of the surrounding landscape, has been underlined [9, 23]. Until now, several approaches have been proposed for assessing the risk of birdstrike at airports [24–27], but only few consider hazardous land-uses on and near airports in the estimation of risk [28–30]. However, these procedures do not quantify the contribution of habitats around airports to birdstrike risk. Here we propose a quantitative tool, named Attraction Risk Index (ARI), which estimates the risk of birdstrike based on the habitat makeup of the airport surroundings. This allows to define how habitat types contribute to risk. We hypothesized that risk of birdstrike at airports is driven by land-uses within a 13-km radius from the runway that strongly affect the abundance and distribution of wildlife in the study area, depending on the time of the year. Therefore, habitats near airports play a significant role in the occurrence of bird-aircraft collisions in terms of impacted species and number of individuals involved. Furthermore, we believe that risk is a function of the flight altitude of birds within the 13-km buffer from the airport, since it determines whether or not they could interfere with aircraft, leading to birdstrike occurrence. Finally, the air traffic and the severity indices associated to impacts are necessary components in our risk metric since they contribute to increase the probability of birdstrike and to provide the effect on flight caused by collisions. Our aims were 1) to provide airport managers a site-specific tool that quantifies the risk of birdstrike at airports and that is sensitive to land-use changes within a 13-km buffer from the runway; 2) to highlight areas in the airport’s surroundings that contribute most to birdstrike risk and provide a decision-making support tool for managing landscape in the proximity of airports; and 3) to define the most hazardous groups of species in which to direct the management efforts. Ultimately, this work aims to provide a standardized approach, applicable on a large scale, since it uses publicly accessible data on bird occurrence in the airport surroundings and on land cover of the study area (i.e. Bird atlases and CORINE Land Cover databases). Such data are thus readily available to all airports. In the event that is not possible to have information on bird presence for the area where the studied airport is located, we tested the opportunity of using models developed for an airport for which such data are available. This was done to estimate the risk of birdstrike on the target airport, based on the habitat makeup of its surroundings.

## Materials and Methods

### Study area and data collection

**Fig 1:**
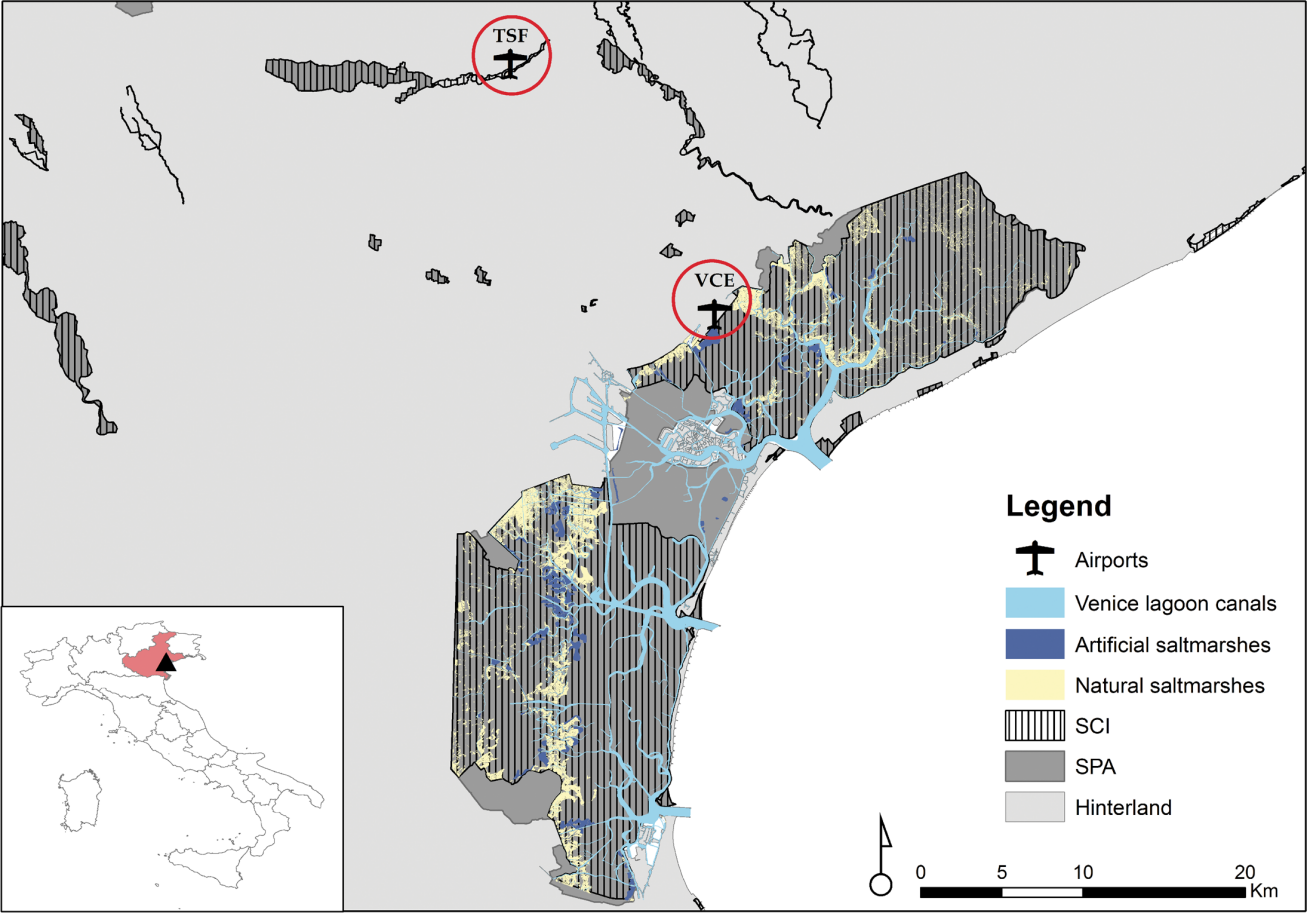
Study area with the two investigated airports Venice Marco Polo (VCE) and Treviso Antonio Canova (TSF). Airports are located in the Northeast of Italy.

Our approach was developed at Venice Marco Polo International airport (VCE), located on the inland border of the Venice lagoon, Italy, and then tested at Treviso Antonio Canova International airport (TSF), which is 20 km inland (Fig 1). According to the guidance provided by the International Civil Aviation Organization (ICAO) in the Airport Planning Manual [31], we considered land-uses present within 13-km from the airfield as hazardous to aviation. Habitats within this radius were classified in six main habitat categories according to the CORINE Land Cover classification, CLC: (1) Agriculture, (2) Anthropized area, (3) Wetlands (including Venice lagoon, artificial and natural lakes and fish processing farms), (4) Industrial area, (5) Landfills and (6) Public green spaces.

**Fig 2:**
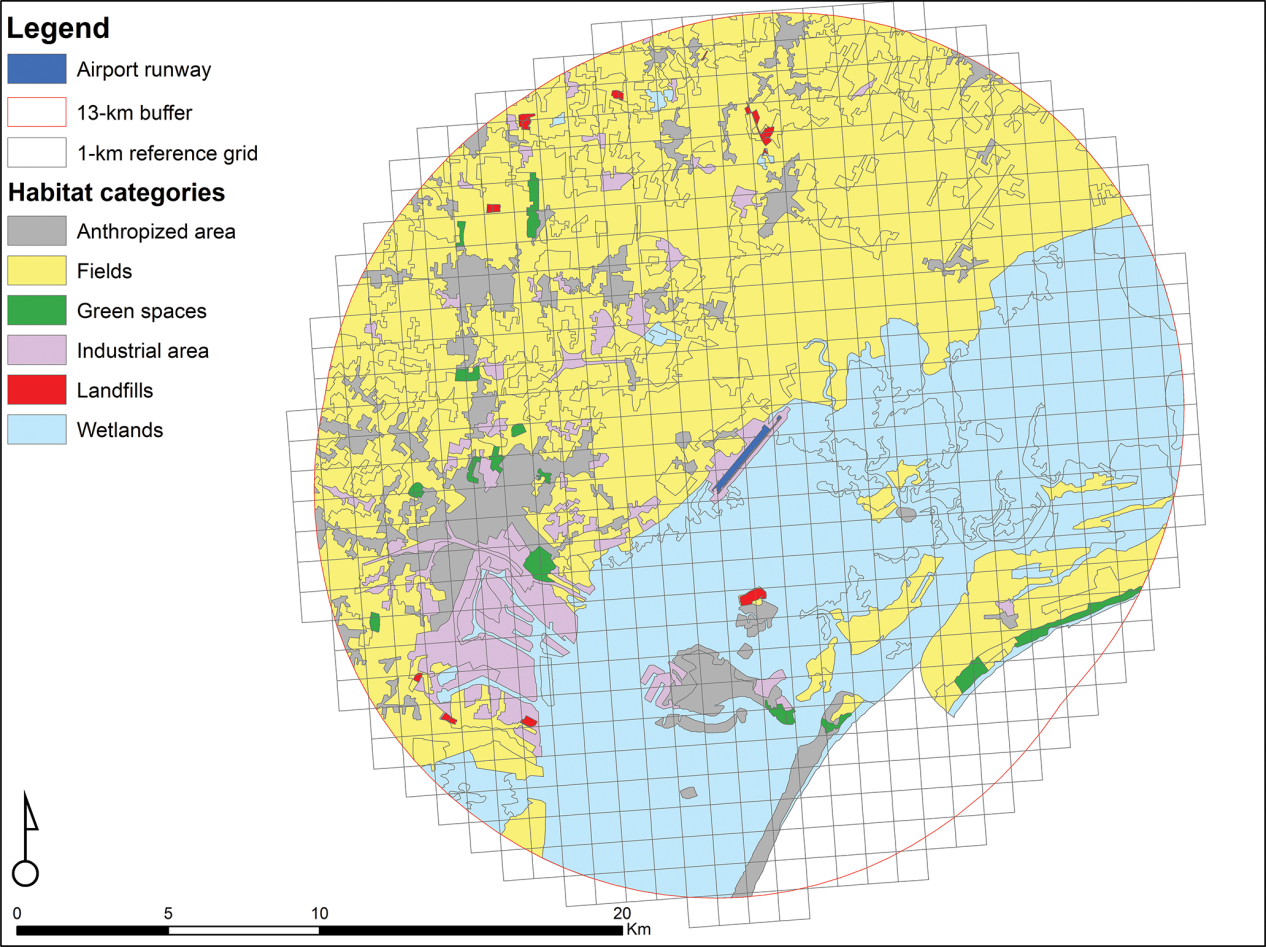
Area within 13-km buffer from Venice Marco Polo airport (VCE) divided in 1-km^2^ cells grid and habitat categories present in it. Land uses were identified using CORINE Land Cover, CLC, classification and mapped using a Geographical Information System, GIS.

Blackwell et al. (2009) [9] encouraged the inclusion of land-use data around airports, as well as wildlife use in these habitats, into wildlife strike risk assessment. In this perspective, we used avian survey data reported in the ornithological atlas of Venice municipality [32], that presents results of bird censuses conducted in the municipality of Venice in the period 2006–2011. For bird atlas data collection, the study area was divided in a regular grid of 1-km cells and, in order to georeference them, entries were assigned to the code of detection unit in which these were recorded. To simplify data management, we classified the recorded bird species according to the group composition proposed by Soldatini et al.(2011) [27]. Furthermore, based on date of surveys, we divided data into four periods, approximately corresponding to the four phases of birds’ biological cycle: wintering (from November to January), spring migration (from February to April), breeding (from May to July) and fall migration (from August to October), to take into account the seasonal variations associated with the ecological needs of birds. For this study, we assumed atlas data as reflective of current conditions in the airport surroundings. Finally, data reorganized as explained above, were analysed in relation to the proportional coverage of the six habitat categories and to distance of cells from the runway (Fig 2 and S1 Dataset). This to estimate the probability of presence per 1-km cell, by groups of species and period of the year, in function of habitat types and position of cell relative to airport. The percentage of habitat categories and distance from the runway were calculated for each cell of the reference grid falling within a 13-km buffer from the airport, using a Geographical Information System, GIS.

Our approach also included data on bird abundance and distribution recorded at VCE airport from 2006 to 2011. Data were collected by professional ornithologists twice a month on an hourly basis from dawn to dusk. In accordance with Soldatini et al. (2010) [33], the airport area was divided into quadrats (370×370 m) and observations were performed from a fixed elevated vantage point from which the study area was clearly visible throughout the year. In addition, during each survey we performed a line transect along the airport perimeter in order to obtain a better estimation of bird community attending the airport area. The transect was 8381 m long and was performed by car (a follow-me airport vehicle) driving at a constant speed of 5 km/h while making counts to a fixed distance of 200 m. The latter survey was conducted at dawn and at dusk, the birds’ peak activity times [34, 35]. Transect data were then used to validate data from vantage point surveys by means of a Spearman Correlation Analysis. Using this perspective, we compared data recorded in the same period of the year and time of the day and collected with the two sampling methods. For the analysis, we considered each phase of birds’ biological cycle and dawn and dusk time slots.

Finally, to develop our risk metric, we used airport traffic data from 2006 to 2011 (i.e. any aircraft take-off or landing at an airport, considering commercial and non-commercial flights) and bird strike data recorded at airport from 2003 to 2011. A bird strike is defined as a collision between a bird and an aircraft in flight, or on a take-off or landing roll. We used bird strike data from three years before the beginning of avian surveys at airport. In fact, this is a relatively recent past, thus not affected to long-term variations in wildlife populations. At the same time, it avoids that biased weight is given to anomalous years, where particularly high, or low, number of impacts was recorded [26]. Furthermore, in order to take into account events occurring during take-off, approach and landing phases, strikes up to 500ft were considered for the analysis. Information on bird-aircraft collisions were recorded by pilots and airport operators according to the Bird strike Reporting Form (BSRF), available in the APT01-B, Annex I [36]. Data on birdstrike events and aircraft movements were provided by the airport management authority SAVE S.p.A. As for data from the ornithological atlas of Venice municipality [32], bird species registered in airport survey and birdstrike databases were grouped according to Soldatini et al. classification [27].

### Attraction Risk Index

Presence/Absence data (P/A) derived from bird atlas of Venice municipality, in relation to the proportional coverage of habitat categories and to the distance from the runway (see S1 Dataset), were used to develop Generalized Linear Models (GLMs) with binomial distribution in order to estimate the probability of presence (PP), by group of species (k) and period of the year (p), per cell of the grid falling within a buffer of 13-km from the airport. We formulated five different models to represent alternative hypotheses on the role of attractants to wildlife use of the area (with * representing the interaction between two terms), assuming the independence of observations (i.e. 1-km cells of the reference grid):

$$\begin{array}{l} \text{m}1)\;\text{P/A}=\text{period}+\varepsilon_{\text{i}};\\ \text{m}2)\;\text{P/A}=\text{Anthropized area}+\text{Fields}+\text{Green spaces}+\text{Industrial area}+\text{Landfills}+\text{Wetlands}+\text{Distance}+\varepsilon_{\text{i}};\\ \text{m}3)\;\text{P/A}=\text{Anthropized area}+\text{Fields}+\text{Green spaces}+\text{Industrial area}+\text{Landfills}+\text{Wetlands}=\text{Distance}+\text{Period}+\varepsilon_{\text{i}};\\ \text{m}4)\;\text{P/A}=\left(\text{Anthropized area}+\text{Fields}+\text{Green spaces}+\text{Industrial area}+\text{Landfills}+\text{Wetlands}+\text{Distance}\right)\;*\;\text{Period}+\varepsilon_{\text{i}};\\ \text{m}5)\;\text{P/A}=\left(\text{Anthropized area}+\text{Fields}+\text{Green spaces}+\text{Industrial area}+\text{Landfills}+\text{Wetlands}+\text{Distance}\right)\;*\;\text{Period}\;*\;\text{Distance}+\varepsilon_{\text{i}}; \end{array}$$

We then used the Akaike Information Criterion, AIC [37], to select the best model (smallest AIC) among the sets of candidates. Furthermore, we estimated the importance of variables for model selected per group of species, through an evaluation of the loss of deviance explained when excluding from the model a covariate at a time. In this way, we could define how different types of habitat influence the wildlife use of the study area. However, the estimated probability of presence, PP, does not necessarily elevate the risk of birdstrike. In fact, the risk is linked to the possibility for birds and aircraft to come in contact. Therefore, the probability of presence of a group of species in a given cell of the grid should be considered only if this group can reach the flight altitude of aircraft in transit over that cell, since only then might it interfere with airplanes. For this purpose, according to the Airport Planning Manual [31], we divided the study area in three buffer zones of 3, 8 and 13 km. In these zones, aircraft fly at a height of (respectively) 0–500, 501–1300 and 1301-2000ft. We then used information on the flight altitudes of birds found in literature [38–40], to generate an indicator variable (I). This indicator variable determines whether or not groups of species could reach the height of flight of aircraft associated to the three buffer zones (Table 1). Multiplying GLMs predictions by this indicator variable allowed us to omit those estimates of PP for which the value of I was equal to zero.

**Table 1:** Indicator variable I which determines whether or not the fifteen groups of species could reach the altitude at which aircraft fly when at 3, 8 and 13 km buffer from the runway.

		Buffer (km) and Height (ft)		
ID group	Species group	3 km (0-500ft)	8 km (501-1300ft)	13 km (1301-2000ft)
1	Grebes and divers	1	1	1
2	Cormorant, pelicans, swans and geese	1	1	1
3	Herons, storks, flamingos	1	1	1
4	Ducks, pheasants, rallids	1	1	1
5	Birds of prey—large	1	1	1
6	Birds of prey—small	1	1	1
7	Seabirds—large	1	1	0
8	Seabirds—small	1	1	0
9	Waders	1	1	1
10	Doves	1	1	0
11	Owls	1	0	0
12	Swifts and swallows	1	1	1
13	Corvids	1	1	1
14	Non-flocking passerines and bats	1	1	1
15	Flocking passerines	1	1	1

We considered two additional factors that contribute to increase the probability of interference between aircraft and birds: the air traffic (AF) and the number of birds crossing the airport airspace (BC). The former is considered as the monthly number of flights recorded at airport. The latter is calculated on data from the airport surveys and is given by the ratio between the number of birds (NB) per group of species recorded at airport in a specific time of the year and the number of surveys (NS) performed in that time (Eq 1). Now, the estimates of probability of presence (PP*I) per cell of the study area were summed by group of species and period of the year. The obtained values were then multiplied by AF and BC variables to obtain the interference prospective between aircraft and birds (IP) (Eq 2).

Ultimately, we included in our risk metric the severity indices associated to bird-aircraft collisions, as strictly involved in risk determination [21, 26]. In this perspective, we considered three variables: the average weight (W) and median flock size (MFS) of groups of species recorded at the airport and the Effect On Flight (EOF) caused by impacts with each group. In accordance with the Advisory Circular APT01-B, Annex 6 [36], the EOF variable is divided into five categories of severity. These range from no effect (EOF = 1) to ‘inability of aircraft restoration’ (EOF = 5). In this study, we used the 95^th^ percentile of the EOF recorded for each group of species, as proposed by Soldatini et al. (2011) [27]. The Attraction Risk Index (ARI) was obtained as the summation over the groups of species of the product between the interference prospective and the severity indices, divided by the mean number of flight per year (mAF). The latter operation is intended to standardize the risk estimation, allowing for comparison among airports (Eq 3).

$$BC^{k}=\frac{\sum NB^{k}}{\sum NS^{k}}$$ Eq 1

$$IP^{k}=BC^{k}\cdot AF\cdot \sum_{1}^{ncell}\left(PP{\left(cell\right)}_{p}^{k}\cdot I_{cell}^{k}\right)$$ Eq 2

$$ARI=\sum_{k=1}^{k=15}\left(\frac{IP^{k}\cdot W^{k}\cdot MFS^{k}\cdot EOF_{95}^{k}}{mAF}\right)$$ Eq 3

### Data analysis

We used the mean value of the probability of presence (PP*I), calculated over the fifteen groups of species, to create risk maps per period of the year and highlight areas that contribute most to birdstrike risk. We computed the ARI risk index at VCE airport for each month from January 2006 to December 2011. We then performed the non-parametric Spearman test to evaluate correlation between ARI and the International Bird Strike Committee strike rate (expressed as strikes per 10,000 aircraft movements), in order to check for the accuracy of our risk estimation. Further, the Spearman test was used to determine possible correlations between the computed ARI group-specific risk and the actual number of strikes and the highest Effect On Flight. In this way, we could evaluate firstly which groups most affect the birdstrike occurrence and severity, and secondly whether the ARI risk ranking for groups of species corresponded to the actual frequency ranking for species struck. Finally, to test the ARI index on airfields with a different habitat makeup in its surroundings, we used GLM models developed for VCE to estimate the probability of presence (PP) at TSF airport. For this purpose we used land-use data within 13-km buffer from it as predictors. These estimates were then used to calculate the ARI risk index at TSF from May 2010 to April 2011 and from January 2012 to June 2014. For the computation of the risk index we used data on airport bird surveys and aircraft movements over 2010–2014 and bird strike data from three years before the beginning of surveys at airport. Therefore, to check the reliability of the approach used, we performed the Spearman test for comparison between the ARI index and the birdstrike rate per 10,000 aircraft movements calculated for TSF. For all statistical tests significance value was set at P<0.05.

## Results

In most cases, the model selection criterion indicated model 5 as the one with the stronger support by the data. Model 1 (in which only periodicity was considered) was selected for groups 1 and 11, for which few presence data were available and for which the percentage of deviance explained was particularly small (S1 Table).

**Fig 3:**
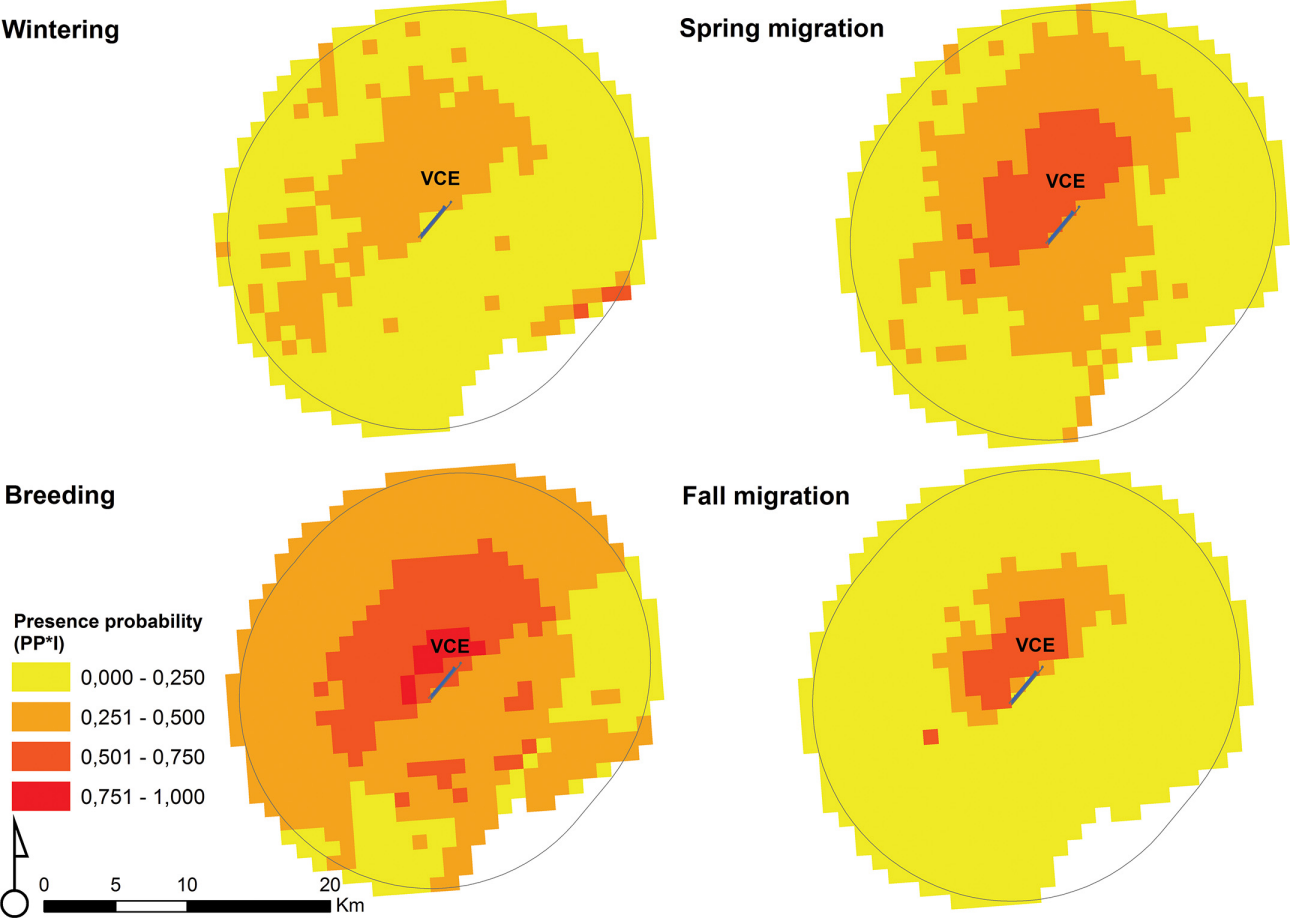
Risk map per period of the year which highlights areas within a 13-km buffer from Venice Marco Polo airport (VCE) that contribute to birdstrike risk. The map shows the mean value of the probability of presence of birds (PP*I) per cell of the reference grid, calculated over the fifteen groups of species.

Developed risk maps pointed out a high probability of bird presence (PP*I ≥ 0.500) in the cells located on the north side of the airport, over the four periods of the year (Fig 3). These cells are mainly represented by agricultural fields (88.02%) and, to a lesser extent, by wetlands (3.30%), industrial areas (3.00%), anthropized areas (2.96%) and public green spaces (2.72%) (the landfill category is missing). On the contrary, areas on the southern part of the airport showed a probability of presence between 0.251 and 0.500 solely during spring migration and breeding periods. These cells are constituted by wetlands (i.e. the lagoon of Venice) for 62.44%, followed by agricultural fields (27.65%), anthropized areas (5.81%), industrial areas (2.52%), public green spaces (1.33%) and landfills (0.25%). Specifically, the estimated importance of covariates for models selected by each group of species indicated fields as the most important habitat for groups 4, 5, 6, 13 and 14 (pheasants, birds of prey, corvids and non-flocking passerines). Wetlands are most attractive for groups 2, 3, 7, 8 and 9 (in particular cormorants, herons, gulls and waders), while urban areas primarily attract groups 10, 12 and 15 (synanthropic species such as feral pigeons and starlings and migratory species) (Table 2). Groups 1 and 11 (grebes and owls) were excluded from the analysis since model 1, which considers only the periodicity, was selected for these groups. Results also highlighted the key role of distance of cells from the runway on the probability of presence of birds. Indeed, for all groups, the lower the distance from the airport, the higher the probability of presence (S1 Fig).

**Table 2:** Model selected per group of species and relative number of parameters (K), Log-Likelihood, Deviance explained and loss of deviance explained by excluding from the model a covariate at a time.

					Loss of Deviance explained					
ID group	model selected	K	Log-Lik	Dev. expl.	Urban area	Fields	Industrial area	Landfills	Wetlands	Distance from runway
1	1	4	-387,100	4,606	0,000	0,000	0,000	0,000	0,000	0,000
2	5	55	-523,818	22,792	-0,032	-0,028	-0,026	-0,010	**-0,056**	-0,065
3	4	32	-663,559	23,382	-0,009	-0,010	-0,006	-0,004	**-0,013**	-0,069
4	5	56	-643,571	25,303	-0,023	**-0,098**	-0,020	-0,032	-0,062	-0,126
5	5	56	-473,524	27,886	-0,031	**-0,071**	-0,023	-0,034	-0,041	-0,141
6	5	56	-429,281	31,581	-0,027	**-0,106**	-0,023	-0,024	-0,053	-0,163
7	5	56	-666,532	28,373	-0,041	-0,063	-0,025	-0,024	**-0,126**	-0,119
8	5	56	-638,714	21,137	-0,024	-0,071	-0,023	-0,023	**-0,109**	-0,116
9	5	55	-553,961	24,919	-0,015	-0,088	-0,010	-0,009	**-0,107**	-0,106
10	4	32	-527,131	43,215	**-0,071**	-0,038	-0,006	-0,015	-0,033	-0,134
11	1	4	-203,151	5,437	0,000	0,000	0,000	0,000	0,000	0,000
12	4	32	-378,284	56,016	**-0,046**	-0,012	-0,007	-0,009	-0,018	-0,164
13	5	56	-553,772	39,621	-0,052	**-0,096**	-0,025	-0,023	-0,050	-0,130
14	5	56	-553,851	41,631	-0,092	**-0,104**	-0,021	-0,020	-0,079	-0,170
15	5	55	-515,318	45,114	**-0,115**	-0,107	-0,031	-0,033	-0,077	-0,188

Habitat covariates contributing most to attract each group of species are highlighted in bold.

**Fig 4:**
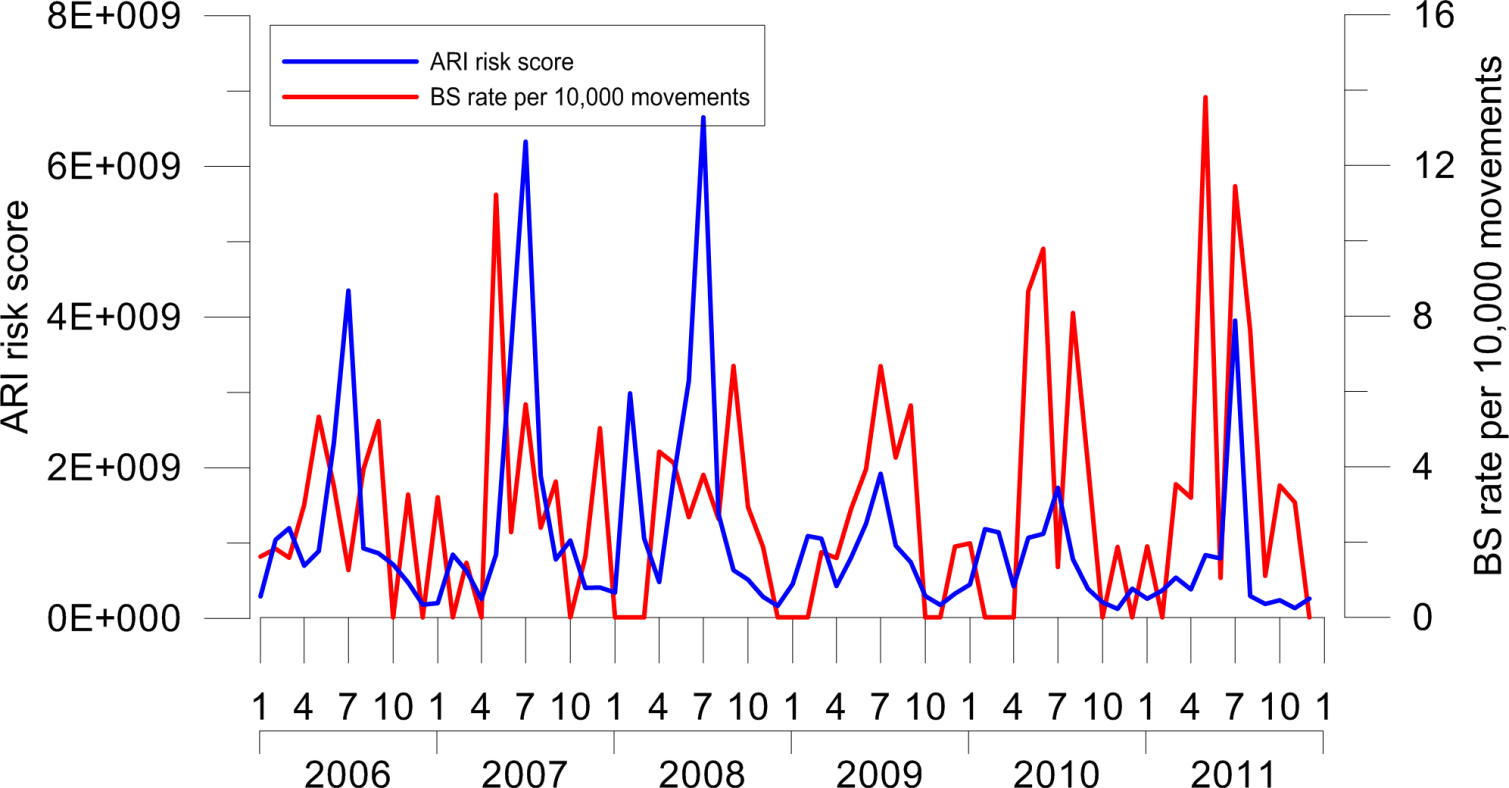
ARI computed for Venice Marco Polo airport (VCE) in the period 2006–2011 compared to birdstrike rate per 10,000 aircraft movements. A clear seasonal pattern of the ARI risk index is visible, with higher values in late summer months. A significant correlation between ARI and the birdstrike rate computed for VCE was found (Spearman test, P<0.05).

The ARI risk index computed for VCE airport showed a clear seasonal pattern with higher values in late summer months. This trend was significantly correlated with the birdstrike rate for VCE airport, which indicates a higher rate in summer and autumn (Spearman test, S = 44798.99, P = 0.017, P<0.05, rho = 0.279) (Fig 4).

Furthermore, we found a significant positive correlation between the computed ARI group-specific risk and the actual number of strikes in the case of groups 3 (Spearman's rank correlation rho = 0.485), 6 (rho = 0.447), 7 (rho = 0.500), 8 (rho = 0.368), 10 (rho = 0.515), 12 (rho = 0.539) and 13 (rho = 0.428). Therefore, according to the ARI risk index, these groups are those contributing most to birdstrike occurrence (S2 Fig). On the other hand, a significant negative correlation was found for group 5 (rho = -0.319). Finally, the ARI group-specific risk was correlated with the highest Effect On Flight for groups 4 (rho = 0.543), 5 (rho = 0.412) and 15 (rho = 0.419), which thus appear as those contributing positively to the severity of strikes. However, since data related to birdstrike events with an EOF >1 were very few, these analysis are yet to be investigated.

**Fig 5:**
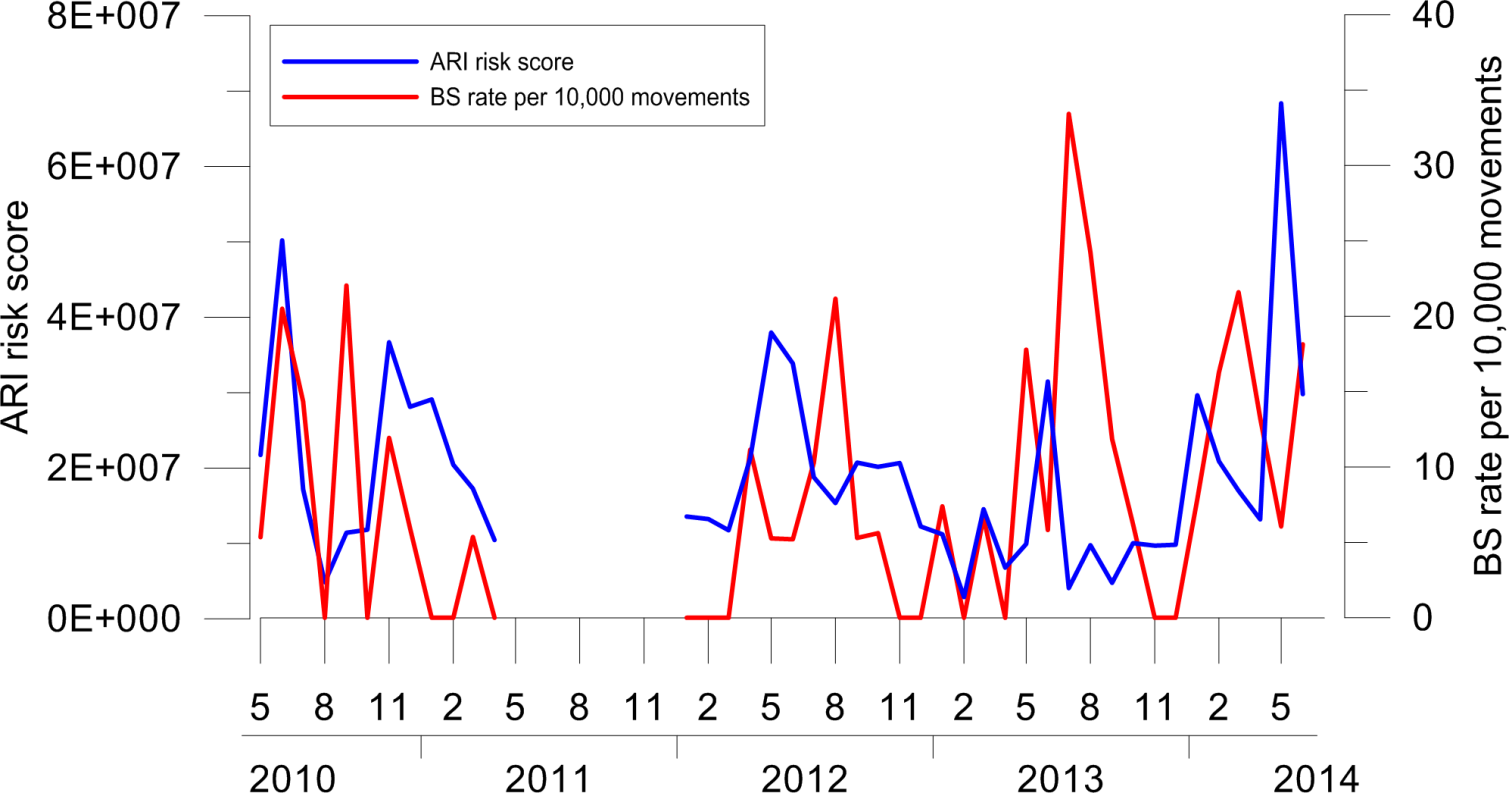
ARI computed for Antonio Canova Treviso airport (TSF) in the period 2010–2014 compared to birdstrike rate per 10,000 aircraft movements. At TSF, ARI shows a different trend compared to VCE, with higher risk scores in summer and winter. No significant correlation between ARI and the birdstrike rate computed for TSF was found (Spearman test, P>0.05).

The ARI risk index computed for TSF airport showed a different trend compared to VCE, with higher risk scores in summer and winter. However, we found no significant correlation between ARI and the birdstrike rate per 10,000 aircraft movements calculated for TSF, with the latter showing higher values in spring and fall migration (Spearman test, S = 10802.18, P = 0.431, rho = 0.124) (Fig 5).

## Discussion

In this study we developed a tool that allows the quantitative estimate of birdstrike risk at airports, based on the habitat types present in its surroundings. Land-uses within a buffer of 13-km from the airport attract birds with a different degree depending on the group of species, the period of the year and their position relative to airport. Therefore, the specific habitat makeup around an airport contribute to birdstrike risk occurrence. Results from the processing of risk maps in a buffer of 13-km from VCE airport indicated that, among the habitat categories present in the study area, agricultural fields attract birds during all phases of their biological cycle (Fig 3). In particular, this habitat type contributes most to the occurrence of groups 4, 5, 6, 13 and 14. Such groups are represented by terrestrial bird species (e.g. *Phasianus colchicus*, *Buteo buteo*, *Falco tinnunculus*, *Corvus corone cornix*, *Turdus merula*), resident in the territory where the airport is located and which exploit fields to feed, shelter and resting. On the contrary, wetlands (i.e. the Venice lagoon) are an attractant solely during spring migration and breeding periods (Fig 3) and are the most important habitat for groups 2, 3, 7, 8 and 9. These groups consist in waterfowls, some of which are migratory species arriving in the study area from mid-March to nest in the lagoon (e.g. *Bubulcus ibis*, *Sterna hirundo*, *Sternula albifrons*, *Haematopus ostralegus*, *Charadrius alexandrinus*). The third habitat category which proved to contribute most to bird occurrence is the anthropized area. This habitat primarily attracts groups 10, 12 and 15. Among the species belonging to these groups, some exploit urban areas throughout the year (e.g. *Columba livia*, a domestic form), registering a maximum peak of breeding attempts in March–July[41]. Others are migratory species attending these areas in breeding season, nesting on buildings, bell towers or churches (e.g. *Hirundo rustica*, *Apus apus*, *Sturnus vulgaris*). Therefore, according to these findings, we would expect to have birdstrike events involving groups 4, 5, 6, 13, 14 throughout the year, while groups 2, 3, 7, 8, 9, 10, 12 and 15 primarily struck in warmer periods. These expectations partially match with the actual frequency ranking for groups of species struck per period of the year (S3 Fig). Furthermore, our results have revealed the key role of cell distance from the runway on probability of bird presence (PP) in the airport surroundings. Indeed, it appears that the shorter the distance from the runway, the higher the probability of presence. This confirms the attractiveness of airports to birds [42–44] and demonstrates how distribution of birds in the territory is affected by the presence of airfields.

At VCE, where the ARI index has been developed, we found a clear seasonal effect with higher risk scores in late summer months (Fig 4). This result emphasizes the importance of considering seasonality in the risk matrix, since its influence on the risk of birdstrike is widely known [45].

ARI was significantly correlated with the birdstrike rate per 10,000 aircraft movements. This suggests the validity of our method. However, relying on airport strike rate as a measure of risk may be simplistic since several factors affect the risk of birdstrike, including land-uses around airport [9]. In addition, a higher number of strikes does not necessarily mean a higher risk (e.g. rare events with a flock of large birds may result in a more severe outcome than more frequent incidents with smaller species). The ARI index fits in this perspective for the estimation of risk. In fact it does not consider the number of impacts occurred in a specific time, but the effect of impact on aviation.

According to ARI, groups 3, 6, 7, 8, 10, 12 and 13 contribute most to birdstrike occurrence at VCE (S2 Fig). From a qualitative comparison between the ARI risk rankings and the actual risk rankings for groups of species struck at VCE, there appears to be a correspondence in four cases of seven (S3 Fig). Also, groups highlighted by ARI match with species most involved in collisions with aircraft on a national scale, which are *Larus michahellis* (group 7), *Falco tinnunculus* (group 6) and *Apus apus* (group 12) [46]. This is further proof of the reliability of the method developed. On the contrary, group 5 of large birds of prey was negatively correlated with the actual number of birdstrikes. Probably, species belonging to this group (e.g. *Buteo buteo* and *Circus aeruginosus*) are affected by the increased air traffic. This leads to an enhanced probability of birdstrike occurrence and, simultaneously, of abandonment the airport area towards safer and less disturbed places.

Again, groups contributing positively to the severity of birdstrike were groups 4, 5 and 15. These groups are characterized by high average weights (W_4_ = 752; W_5_ = 805 grams) and/or high median flock size values (MFS_4_ = 23; MFS_15_ = 49 no. of individuals). Therefore, impacts with these groups may result in serious outcomes, leading to a higher risk. However, the distribution of birdstrike events with a significant effect on flight (EOF_95_>1) was sporadic in the dataset. This calls into question the reliability of outcomes: to validate a risk index, like the one proposed in this study, a reliable measure of the effects caused by impact is needed. In our opinion, to assess the contribution of groups of species to the severity of bird-aircraft collisions, it would be more appropriate to move from a categorical variable as it is currently classified the effect on flight [36], to a continuous variable based for example on the cost per strike.

At TSF, the ARI index showed a different seasonal pattern from that outlined for VCE. There were higher risk scores in summer and, in fewer cases, in winter (Fig 5). This pattern is based on the probability of bird presence within a buffer area of 13-km from the airport (S4 Fig), which depends on the habitat makeup of the study area (S5 Fig). In particular, it appears that in the breeding period, agricultural fields and wetlands (i.e. lakes and fish farms) contribute most to the occurrence of birds in the area, while in winter landfills are most attractive. Landfills were mainly attractive for groups 7, 8 and 13 (i.e. gulls and corvids). This result coincides with what is reported in literature. In fact, the use of food sources resulting from human activities (e.g. landfills, fishery bycatch, sewage outfalls and slaughterhouses), especially in adverse season, is widely known for synantropic species [47–49].

Our findings for TSF airport show that ARI is actually driven by land-uses in the airport surroundings. The findings also highlight the plasticity of ARI in adapting to the morphology of the area on which the studied airport is located. However, we found no significant correlation between the ARI index and the birdstrike rate computed for TSF. This reveals that use of GLMs developed for VCE to estimate the risk of birdstrike at TSF lowers the effectiveness of the index and underlines the importance of using site-specific data for the computation of ARI.

Despite the great importance given to management of resources on and near airports to reduce the risk of birdstrike, as recently highlighted by some Authors [50], until now no method has incorporated habitats around an airport in the estimation of risk. Our index represents a first step towards filling this gap. We provide information on the contribution of habitats to birdstrike risk, by group of species and period of the year. These findings can be used by airport managers and local authorities to plan specific interventions in the study area.

For this study, we used data on bird occurrence and land cover of the study area from publicly available sources (i.e. ornithological atlas of Venice municipality and CORINE Land Cover) as we aimed to develop a standardized tool (Bird atlases and CLC use a standard approach for data collection) that was usable by all airports and applicable on a large scale. However, where possible, the use of data collected specifically for the purpose of the study (i.e. data on bird occurrence specific per land-use categories in a buffer of 13-km from the airport) and following an objective sampling protocol is desirable, as it increases the accuracy of ARI risk estimates. Also, surveys on airports must be designed using a standardized protocol in order to yield accurate estimates of species abundances, since they are critical to birdstrike risk. This issue has been recently addressed by Blackwell et al. (2013) [20]. In our study, we used transect data to validate data from vantage point surveys. In fact, the latter method of sampling may result in detection bias, since detectability of birds to human observers declines with distance [51]. However, results from the correlation analysis between data from the two survey techniques revealed a significant correlation in all cases, except for the breeding period in dusk time (S2 Table). These findings validate the use of data from a fixed point observation for our analysis.

Future research will be aimed to test our approach on different case study airports in order to include a broader variability of territorial conditions and increase the generality of the developed method. Moreover, data will be collected specifically for the scope of the study, using rigorous avian survey techniques. This to maximize the accuracy in the quantification of use of airport and near-airport habitats by bird populations. In this way we could evaluate whether the quality of the ARI index increases as a result of these changes.

## Supporting Information

S1 Dataset Avian survey data from the ornithological atlas of Venice municipality classified by the fifteen groups of species and the four periods of the year and recorded per cell of the reference grid. Data are related to the proportional coverage of the six habitat categories selected for this study and to the distance of the centroid of cells from the runway.
(XLSX)

S1 Table AIC scores and Akaike weights (ωAIC) for the five developed models and model selected among the set of candidates (smallest AIC). (XLSX)

S2 Table Spearman correlation analysis between vantage point data and transect data considering the four phases of birds’ biological cycle and dawn and dusk time slots. (XLSX)

S1 Fig Probability of presence (PP), by group of species, estimated per cell of the reference grid within a 13-km buffer from Venice Marco Polo airport (VCE). (TIFF)

S2 Fig ARI group-specific risk compared to the actual number of strikes recorded at Venice Marco Polo airport (VCE) from 2006 to 2011. According to ARI, groups 3, 6, 7, 8, 10, 12 and 13 contribute most to birdstrike occurrence.
(TIFF)

S3 Fig Stacked bar plot of birdstrike events recorded at Venice Marco Polo airport (VCE) from 2003 to 2011 by group of species and coloured per period of the year. (TIFF)

S4 Fig Risk map per period of the year which highlights areas within a 13-km buffer from Treviso Antonio Canova airport (TSF) that contribute to birdstrike risk. (TIFF)

S5 Fig Area within 13-km buffer from Treviso Antonio Canova airport (TSF) divided in 1km^2^ cells and habitat categories present in it. (TIF)
