# Supplementary figures and images for: A Land-Use Perspective for Birdstrike Risk Assessment: The Attraction Risk Index
Source: PLoS One. 2015 Jun 26;10(6):e0128363. doi: 10.1371/journal.pone.0128363 (PMC4482575; doi:10.1371/journal.pone.0128363)

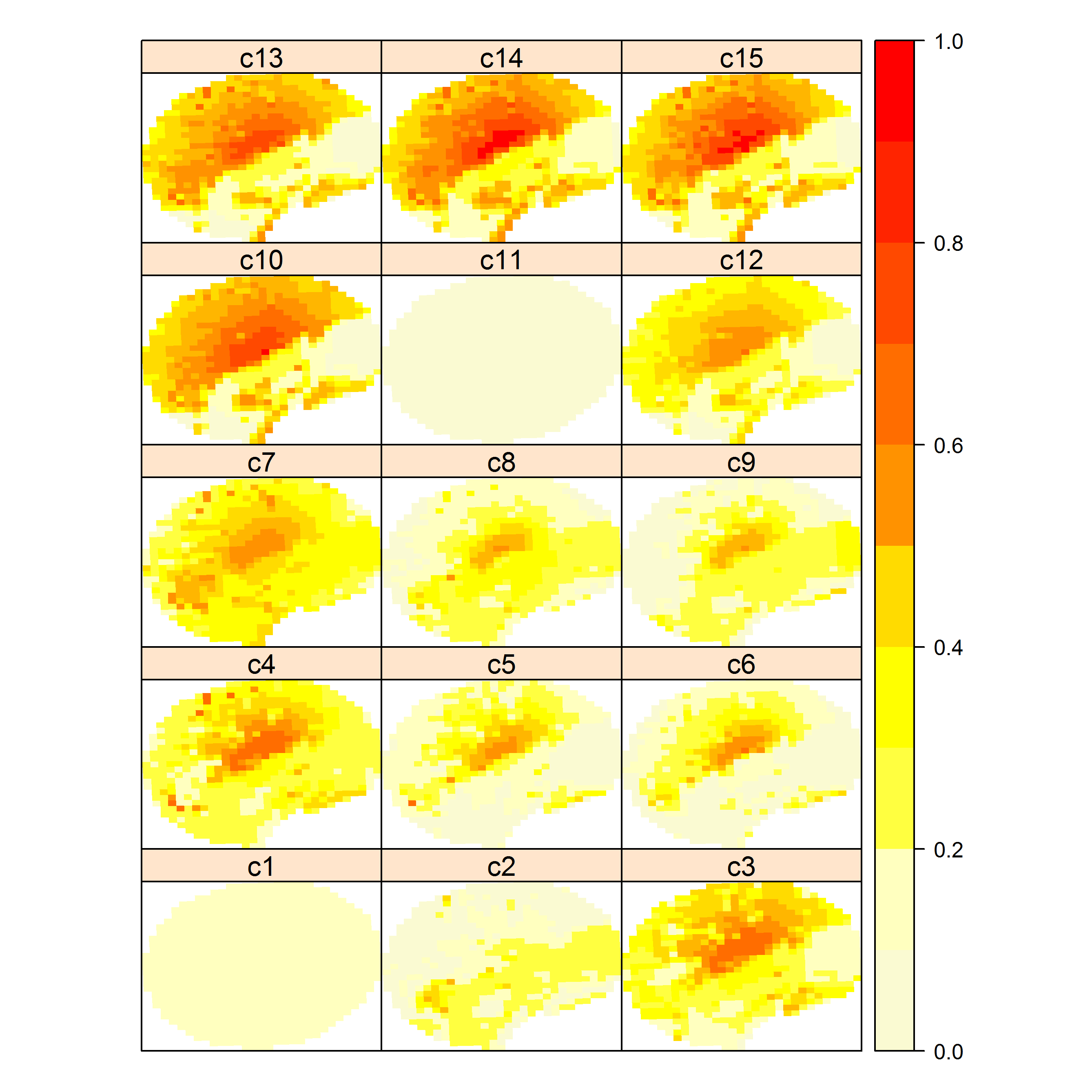

Supplement: S1 Fig — (TIFF) [file pone.0128363.s004.tiff]

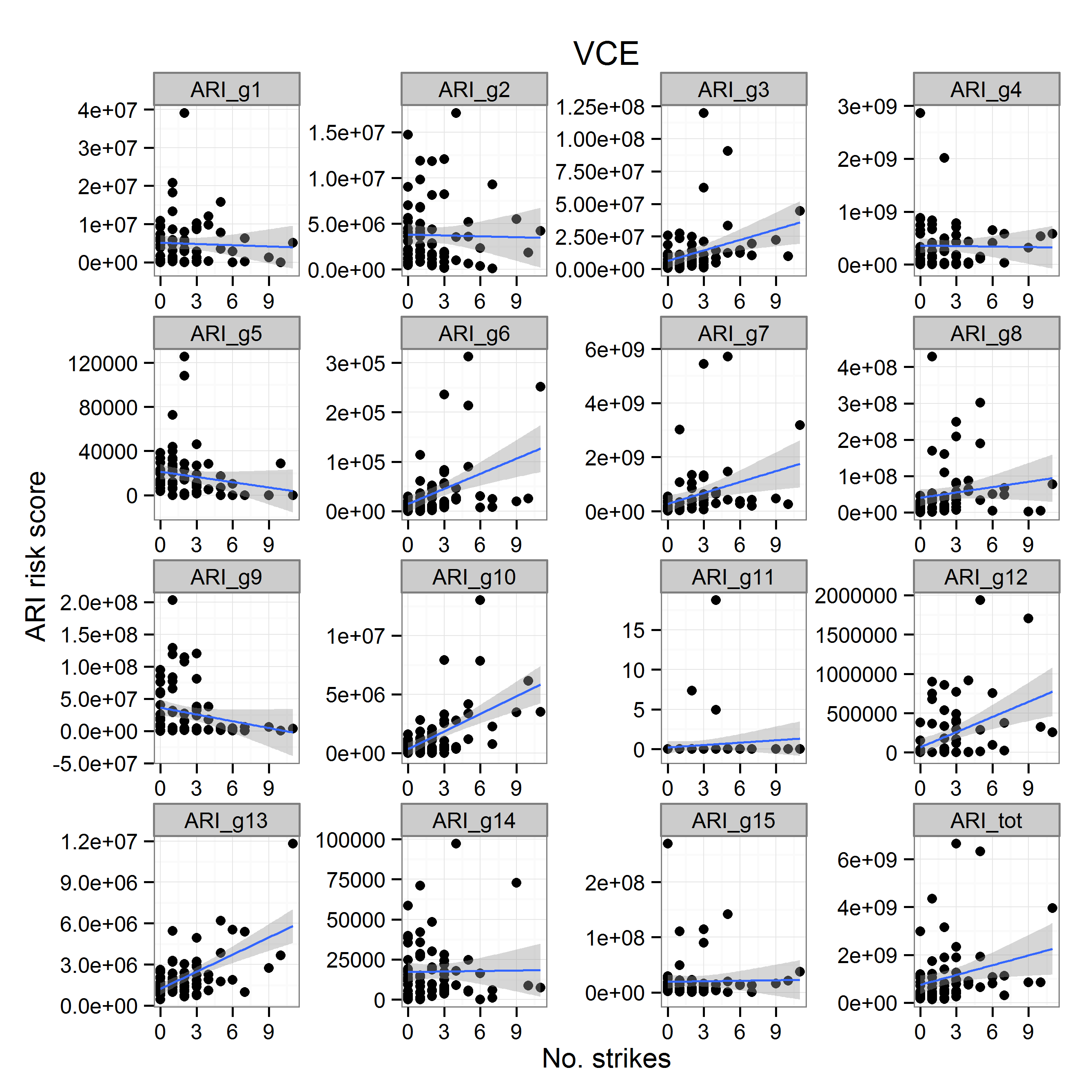

Supplement: S2 Fig — According to ARI, groups 3, 6, 7, 8, 10, 12 and 13 contribute most to birdstrike occurrence. (TIFF) [file pone.0128363.s005.tiff]

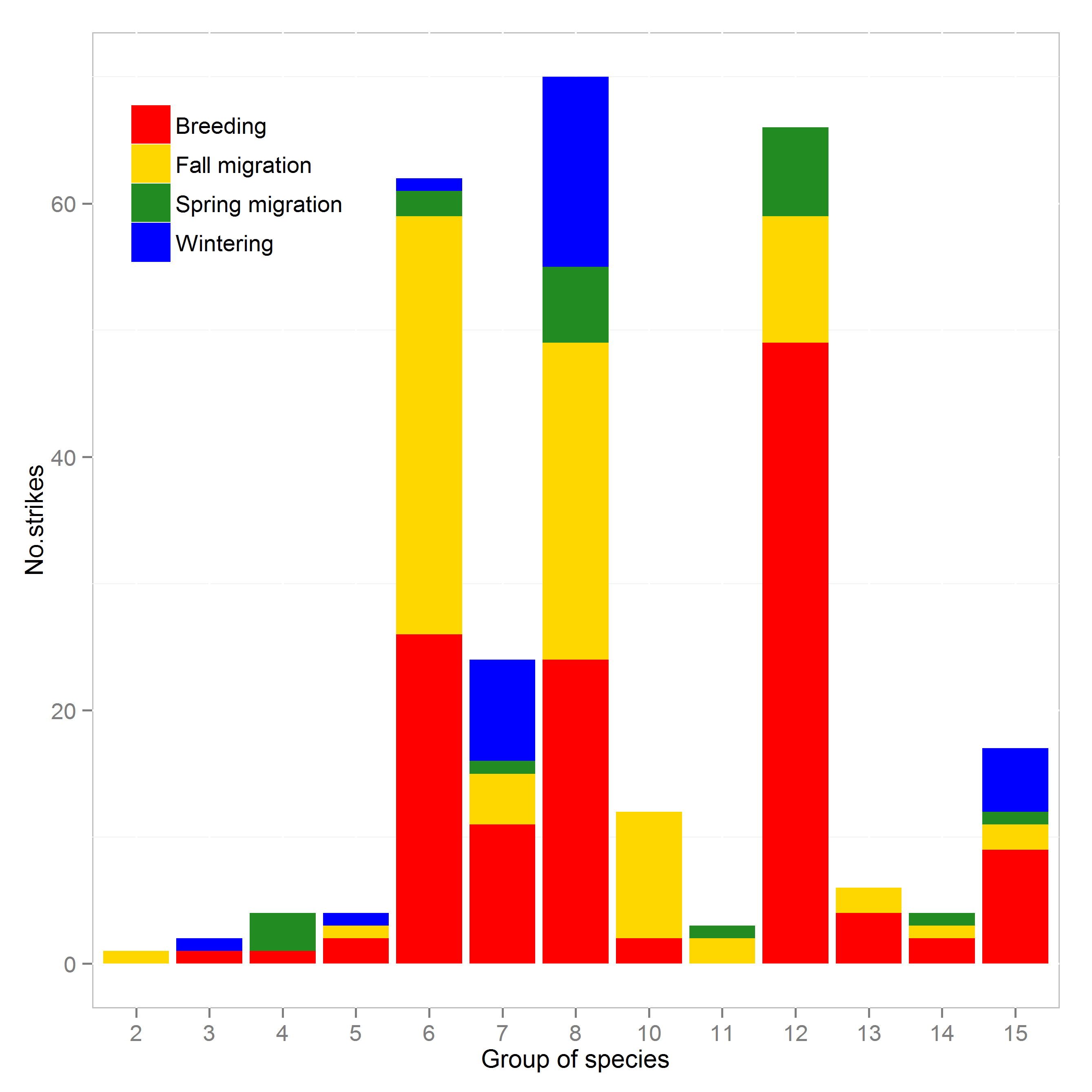

Supplement: S3 Fig — (TIFF) [file pone.0128363.s006.tiff]

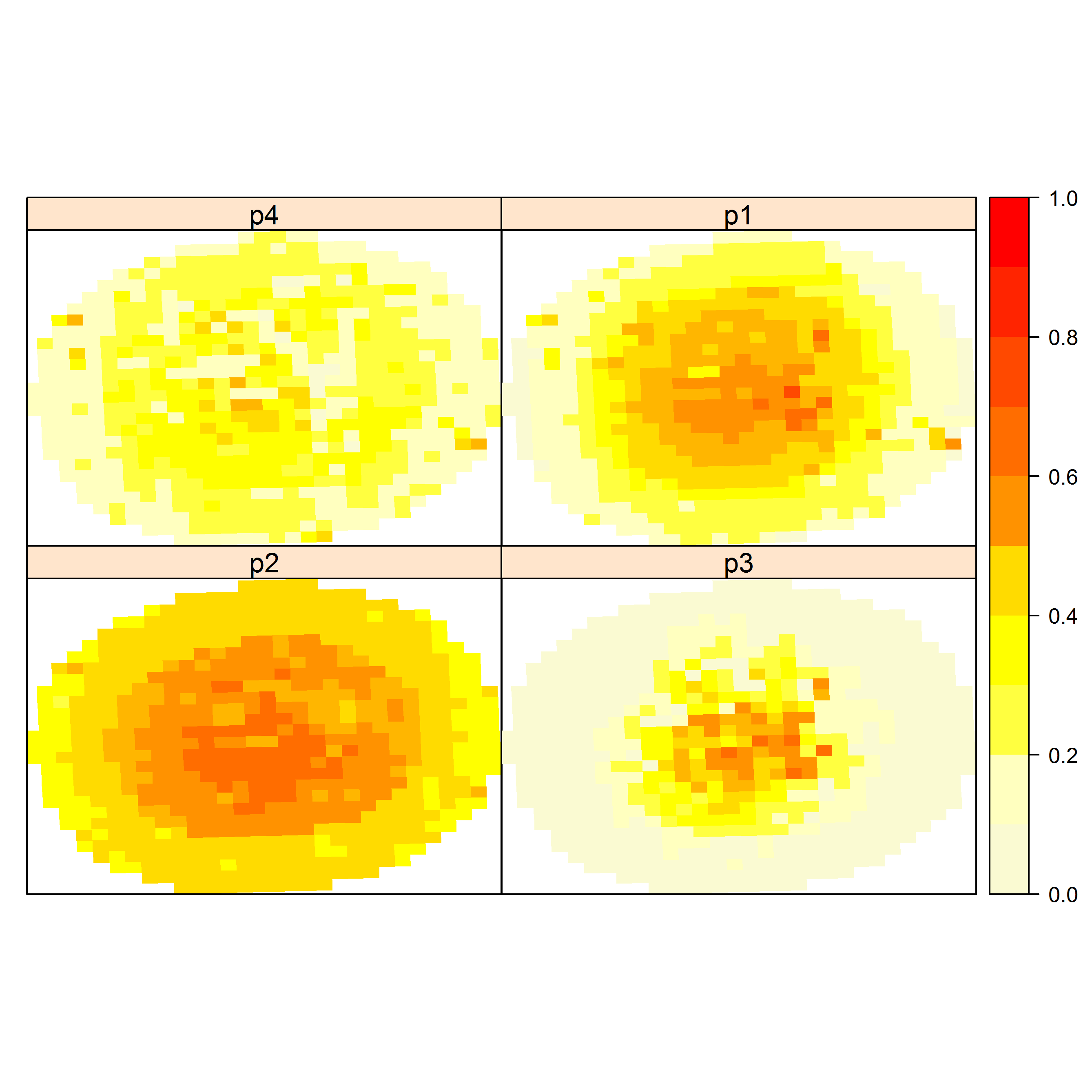

Supplement: S4 Fig — (TIFF) [file pone.0128363.s007.tiff]
